# Triglyceride-glucose index is associated with the severity of coronary artery disease

**DOI:** 10.1371/journal.pone.0356426

**Published:** 2026-08-17

**Authors:** Di Wang, Ying Wang, Fangfang Li, Zhenyu Wang, Kangde Zeng, Shujun Yan, Danyang Liu, Degen Pei, Ling Weng, Enze Jin

**Affiliations:** 1 The Fourth Affiliated Hospital of Harbin Medical University, Harbin, Heilongjiang, China; 2 Pathology Laboratory, Harbin Medical University, Harbin, Heilongjiang, China; 3 Laboratory of the Second Affiliated Hospital of Harbin Medical University, Harbin, Heilongjiang, China; Università degli Studi di Milano, ITALY

## Abstract

Cardiovascular disease(CVD) remains a leading cause of mortality worldwide. Among CVDs, coronary heart disease (CHD) is the most prevalent and represents the primary contributor to chronic disease-related deaths globally. The triglyceride-glucose (TyG) index has been shown to correlate significantly with both mortality and cardiovascular events, indicating its potential as an independent prognostic marker for cardiovascular outcomes. This study aimed to investigate variations in the TyG index among patients stratified by disease severity using Gensini scores and to evaluate the association between the TyG index and disease severity, as well as its diagnostic efficacy in combination with serum biomarkers. A total of 861 patients diagnosed with CHD who underwent coronary angiography were included. All participants were admitted to the Fifth Ward, Department of Cardiovascular Medicine, at the Fourth Affiliated Hospital of Harbin Medical University between June 2022 and June 2024. Comprehensive data, including demographics, laboratory findings, and medical history, were collected from medical records. Based on the Gensini scores derived from coronary angiography, patients were categorized into three groups—low, moderate, and high stenosis—representing the severity of coronary artery occlusion. Multivariate logistic regression analysis was performed to assess the relationship between the TyG index and coronary artery disease severity. TyG levels differed significantly between the mild and severe stenosis groups (p = 0.0021), and between the moderate and severe groups (p = 0.0173). The TyG index, treated as a continuous variable, was significantly associated with coronary stenosis (odds ratio [OR] = 1.591; 95% confidence interval [CI]: 1.237–1.866). After adjusting for confounding factors including age, sex, diabetes, and hypertension, individuals in the second, third, and fourth TyG quartiles exhibited higher odds ratios for severe coronary stenosis compared to the reference group (OR = 0.932; 95% CI: 0.642–1.351; OR = 1.045; 95% CI: 0.691–1.581; OR = 1.101; 95% CI: 0.605–2.004). These findings indicate that the TyG index is positively associated with the extent of coronary artery stenosis and may serve as an indicator of atherosclerosis severity.

## Background

Cardiovascular disease (CVD) remains the leading global cause of death, exerting immense pressure on public health systems and individual well-being [1,2]. In the early 20th century, CVD accounted for less than 10% of global deaths. By the early 21st century, this figure had risen to nearly 50% in developed countries and approximately 25% in developing nations. By 2020, it was projected to cause around 2.5 million deaths annually, further consolidating its status as the foremost cause of mortality worldwide [3–5]. Over the past three decades, the global burden of CVD has steadily increased. In China, it is one of the leading causes of death, accounting for approximately 40% of total fatalities [6,7].

Coronary heart disease (CHD), the most common form of CVD, is the primary cause of death from chronic diseases on a global scale [8]. The pathophysiological basis of CHD is atherosclerosis, which is widely recognized as a chronic inflammatory condition associated with dysregulation in glucose and lipid metabolism [9]. Metabolic disturbances—such as elevated glucose levels, increased insulin resistance, and elevated free fatty acid concentrations—contribute to the progression of atherosclerosis by promoting oxidative stress in vascular endothelial cells and the formation of advanced glycation end products [10].

Studies have shown that high levels of triglyceride-rich lipoproteins lead to the production of oxidized fatty acids, which trigger the release of cytokines, interleukins, and pro-atherogenic adhesion molecules. This cascade induces localized vascular inflammation and accelerates atherosclerotic plaque formation [11]. The TyG index, which combines fasting triglyceride and glucose levels, has emerged as a surrogate marker for insulin resistance and shows a stronger association with cardiovascular events than traditional indicators. Insulin resistance is implicated in endothelial dysfunction [12,13] and contributes to the formation of unstable atherosclerotic plaques [14]. It is also a key factor in the development of both type 2 diabetes and CVD.

The TyG index is increasingly recognized as a reliable marker for assessing insulin resistance [15]. A large prospective cohort study involving 141,243 individuals from five continents demonstrated that the TyG index significantly correlates with cardiovascular mortality and event risk [16]. Elevated TyG levels are associated with an increased likelihood of major cardiovascular events in patients with diabetes and acute coronary syndrome (ACS), underscoring its potential as an independent prognostic biomarker for CHD outcomes [17,18].

Given these findings, identifying risk factors associated with CHD is essential for disease prevention and management. While current therapies primarily focus on lowering low-density lipoprotein cholesterol (LDL-C), even with the advent of PCSK9 inhibitors, a plateau may have been reached in reducing CVD risk among patients with type 2 diabetes and dyslipidemia [19].

In the present study, we focus on a composite index derived from serum triglyceride and glucose levels—the TyG index—to comprehensively evaluate CHD risk factors. This approach enables a more integrated assessment and enhances early detection capabilities. In patients with established CHD, the TyG index provides valuable insight into disease severity. Elevated TyG levels may reflect more complex coronary lesions and a higher risk of adverse cardiovascular outcomes, thereby offering critical reference data for clinical decision-making and prognostic evaluation.

Coronary angiography is considered the gold standard for diagnosing CHD. The Gensini Score (GS) is a widely accepted scoring system used in coronary angiography to assess the severity of coronary artery stenosis [20]. Originally developed by Goffredo G. Gensini in 1975, this scoring method evaluates the complexity of CHD by incorporating three key elements related to coronary artery lesions: the degree of luminal narrowing, a regional weighting factor, and an adjustment for collateral circulation [21].

Stenosis severity is classified in percentage intervals, with a score of 1 assigned to 1–25% stenosis, and the score doubling with each additional 25% increment. Additionally, the GS integrates weighting coefficients based on the myocardial territory supplied by each coronary artery or its branches. Each lesion score is multiplied by an anatomically determined factor reflecting the functional significance of the affected vascular region. The final Gensini Score is calculated by summing all individual lesion scores, thereby providing a comprehensive evaluation of the overall burden of coronary artery disease in a given patient [22].

However, the association between the severity of coronary artery stenosis and the triglyceride-glucose (TyG) index remains inadequately explored. Therefore, the present study aimed to evaluate the correlation between the TyG index and the degree of coronary artery stenosis, as quantified by the Gensini Score, in patients diagnosed with CHD. Specifically, we sought to investigate variations in the TyG index across different levels of stenosis severity based on Gensini scores and to assess the diagnostic efficacy of the TyG index, particularly in combination with serum biomarkers.

## Methods

### Study population and design

This study was designed as a cross-sectional observational analysis conducted in accordance with the Declaration of Helsinki. No data related to patient identity or personal privacy were collected. The study enrolled patients admitted to the Fifth Ward, Department of Cardiovascular Medicine, at the Fourth Affiliated Hospital of Harbin Medical University, who underwent coronary angiography between June 2022 and June 2024.

Inclusion criteria were as follows: (1) adults aged 18 years or older; (2) a diagnosis of hypertension, defined as a history of elevated blood pressure, current use of antihypertensive medications, or systolic blood pressure (SBP) ≥ 140 mmHg or diastolic blood pressure (DBP) ≥ 90 mmHg; Blood pressure was measured by a Yuwell instrument, model YE660D. (3) diabetes mellitus, defined as a fasting blood glucose level ≥ 7.0 mmol/L, a 2-hour postprandial glucose level ≥ 11.1 mmol/L during an oral glucose tolerance test (OGTT), or a random blood glucose level ≥ 11.1 mmol/L.

Critical laboratory reference values included: LDL-C ≥ 3.37 mmol/L, HDL-C in the range of 1.04–1.55 mmol/L, total cholesterol (TC) ≥ 5.18 mmol/L, and triglycerides (TG) in the range of 0.56–1.07 mmol/L. Lipoprotein(a) was considered elevated when exceeding 30 mg/dL. Serum creatinine (Cr) was measured using an automatic biochemical analyzer based on the photometric colorimetric method, with a reference range of 58–110 μmol/L. (4) All participants were required to have undergone coronary angiography in accordance with the study protocol.

Exclusion criteria were: (1) contraindications to coronary angiography or inability to participate in vascular function testing; (2) presence of acute infections, severe arrhythmias, pregnancy or lactation, or major hematological and endocrine disorders; and (3) incomplete clinical data or failure to undergo coronary angiography.

### Ethics statement

The study protocol was approved by the Ethics Committee of the Second Affiliated Hospital of Harbin Medical University (Ethics approval number: ky2020−077). Written informed consent was obtained from all participants, indicating their understanding of the study’s purpose, objectives, and potential risks and benefits. The investigators had access to personally identifiable information during and after data collection. Data access occurred between June 2024 and October 2024 for research purposes.

### Data collection and grouping

Essential clinical data collected for each participant included sex, age, height, weight, systolic and diastolic blood pressure, and medical history regarding smoking, alcohol consumption, hypertension, diabetes, and other relevant conditions. Before assessment, patients were allowed to rest quietly for at least 10 minutes. Blood pressure was measured three consecutive times using an electronic sphygmomanometer, and the average of the three readings was recorded.

Fasting venous blood samples were collected from the antecubital vein in the early morning before breakfast to measure glucose levels and other biomarkers. Coronary angiography data were obtained simultaneously. Body mass index (BMI) was calculated as weight (kg) divided by height squared (m²). Gensini score calculation criteria are detailed in S1 Table.

Participants were divided into quartiles based on TyG index values as follows: Q1 (n = 216, TyG index ≤ 8.49), Q2 (n = 218, 8.49 < TyG index ≤ 8.85), Q3 (n = 212, 8.85 < TyG index ≤ 9.27), and Q4 (n = 215, TyG index > 9.27). Additionally, patients were categorized into three groups based on Gensini scores using a tripartite classification system: mild stenosis group (G1, n = 290, GS ≤ 20), moderate stenosis group (G2, n = 299, 20 < GS < 52), and severe stenosis group (G3, n = 272, GS ≥ 52). Gensini scoring criteria are presented in S1 Table.

### TyG index calculation

The TyG index was calculated using the formula: TyG index = Ln [TG (mg/dL) × FBG (mg/dL)/ 2], where fasting blood glucose (FBG) refers to the glucose concentration measured after an overnight fast of 8–10 hours (water allowed). Triglyceride measurements also required at least 8 hours of fasting and were preferably conducted in the morning.

Participants were instructed to avoid alcohol, high-fat foods, and strenuous physical activity on the day prior to testing. Venous blood samples (1–2 mL) were collected—typically from the antecubital vein—using a sterile disposable syringe. The venipuncture site was disinfected with alcohol prior to sample collection. Fasting blood glucose and triglyceride levels were measured using an automatic biochemical analyzer based on the photometric colorimetric principle.

### Statistical analysis

Categorical variables in the baseline dataset were expressed as absolute numbers and percentages. The normality of continuous variables was assessed using the Kolmogorov–Smirnov (K–S) test. For variables with a normal distribution, means and standard deviations (SD) were calculated; for those with a skewed distribution, medians and interquartile ranges (IQR) were used. Group differences were evaluated using the Kruskal–Wallis non-parametric test. Spearman’s rank correlation coefficient was employed to assess associations between the TyG index, Gensini score, and other relevant clinical variables. Multivariate logistic regression analysis was conducted to identify independent risk factors. The predictive efficacy of the TyG index for detecting coronary artery stenosis was assessed using receiver operating characteristic (ROC) curve analysis, with the area under the curve (AUC) serving as a measure of diagnostic performance. All statistical analyses were two-tailed and performed using SPSS software, version 21.0 (SPSS Inc., Chicago, IL, USA). A p-value < 0.05 was considered statistically significant.

## Results

### Clinical and biochemical characteristics according to TyG index quartiles

Baseline characteristics of the patient cohort are summarized in Table 1. Among the 861 patients enrolled, a significant positive correlation was observed between the TyG index and several physiological and biochemical indicators, including BMI, diabetes, TG, TC, and LDL-C (P < 0.001). In contrast, the TyG index was negatively correlated with age (P = 0.013) and high-density lipoprotein cholesterol (HDL-C) (P < 0.001). Furthermore, patients in higher TyG quartiles exhibited significantly increased Gensini scores.

**Table 1:** Baseline characteristics of patients stratified by TyG index.

Variable	Q1(N = 216)	Q2(n = 218)	Q3(n = 212)	Q4(n = 217)	P value
Age, years(IQR)	66.00(59.00.70.00)	64.00(58.00.71.00)	64.00(57.25,70.00)	63.00(54.00,69.00)	0.011
Male	150(69.4%)	134(61.5%)	133(62.7%)	141(65.6%)	0.314
BMI, kg/m2	24.11 ± 3.60	25.24 ± 3.27	25.42 ± 3.61	26.06 ± 3.30	<0.001
SBP, mmHg	133.48 ± 18.94	134.48 ± 20.53	136.62 ± 20.60	133.62 ± 20.30	0.389
DBP, mmHg (IQR)	80.00(74.00.88.75)	81.00(74.00.89.25)	81.00(75.00,89.00)	82.00(74.00,90.00)	0.783
Smoker Yes	77(35.7%)	82(37.6%)	67(31.6%)	64(29.8%)	0.288
Drinker Yes	25(11.6%)	29(13.3%)	33(15.6%)	24(11.2%)	0.515
Hypertension	120(55.6%)	142(65.1%)	132(62.3%)	140(65.1%)	0.133
Diabetes	30(18.9%)	54(24.8%)	60(28.3%)	106(49.3%)	<0.001
FPG, mmol/L (IQR)	5.08(4.68.5.55)	5.39(4.98,5.97)	5.76(5.16,6.69)	7.067(5.74,8.96)	<0.001
TC, mmol/L (IQR)	3.58(2.98,4.31)	4.01(3.33,4.78)	4.36(3.53,5.28)	4.50(3.66,5.44)	<0.001
TG, mmol/L (IQR)	0.94(0.79,1.06)	1.36(1.18,1.43)	1.84(1.54,2.11)	2.74(2.31,3.50)	<0.001
LDL, mmol/L (IQR)	1.81(1.37,2.26)	2.12(1.52,2.76)	2.31(1.68,3.10)	2.17(1.58,2.95)	<0.001
HDL, mmol/L (IQR)	1.10(0.94,1.27)	1.07(0.91,1.20)	1.03(0.89,1.17)	0.93(0.83,1.05)	<0.001
Lp(a), mmol/L (IQR)	16.00(8.33,36.23)	15.75(7.88,30.23)	14.95(7.45,33.43)	11.40(5.70,27.50)	0.027
Cr, umol/L (IQR)	70.8(61.65,81.48)	72.45(61.58,83.98)	68.30(58.93,79.38)	73.00(60.50,89.4)	0.069
TyG, (IQR)	8.26(8.11,8.39)	8.67(8.58,8.76)	9.06(8.95,9.18)	9.64(9.42,9.92)	<0.001
Gensini (IQR)	28.00(14.00,55.50)	29.50(11.00,60.25)	32.00(14.63,59.00)	45.00(19.00,73.00)	0.001

Data are presented as IQR, mean ± SD, or n (%).

Abbreviations: BMI, body mass index; SBP, systolic blood pressure; DBP, diastolic blood pressure; FPG, fasting plasma glucose; TC, total cholesterol; TG, triglycerides; HDL-C, high-density lipoprotein cholesterol; LDL-C, low-density lipoprotein cholesterol; Cr, serum creatinine concentration; TyG, triglyceride-glucose index; Lp(a), lipoprotein(a).

### Association between TyG index and severity of coronary artery stenosis

According to the baseline characteristics stratified by Gensini score (Table 2), 290 patients were classified into the mild stenosis group, 299 into the moderate group, and 272 into the severe group. The proportion of male patients increased across these groups: 57.00% (165) in the mild group, 64.55% (193) in the moderate group, and 73.53% (200) in the severe group. Patients with severe stenosis (Gensini score ≥ 52) exhibited significantly higher TyG index values, fasting blood glucose levels, serum creatinine concentrations, and increased prevalence of diabetes and hypertension. Additionally, smoking rates were notably higher in this group.

**Table 2:** Baseline characteristics of patients with varying severity of coronary artery stenosis.

Variable	G1(N = 290)	G2(n = 299)	G3(n = 272)	P value
Age, years(IQR)	64.00(58.00.70.00)	64.00(57.00.70.00)	65.00(57.00,71.00)	0.727
Male	165(56.9%)	193(64.6%)	200(73.5%)	<0.001
BMI, kg/m2	24.89 ± 3.63	25.29 ± 3.57	25.44 ± 3.30	0.109
SBP, mmHg	135.56 ± 20.52	134.51 ± 19.00	133.48 ± 20.85	0.649
DBP, mmHg (IQR)	82.00(75.00.90.00)	81.00(75.00.89.00)	80.00(73.00,89.00)	0.230
Smoker Yes	92(31.7%)	102(34.1%)	96(35.3%)	0.658
Drinker Yes	45(15.5%)	35(11.7%)	31(11.4%)	0.260
Hypertension	167(57.6%)	182(60.9%)	184(67.7%)	0.044
Diabetes	54(18.6%)	91(30.4%)	105(38.6%)	<0.001
FPG, mmol/L (IQR)	5.37(4.93.6.01)	5.39(5.00,5.96)	5.81(5.12,7.56)	<0.001
TC, mmol/L (IQR)	4.39(3.63,5.30)	3.92(3.20,4.80)	3.86(3.12,4.73)	<0.001
TG, mmol/L (IQR)	1.47(1.13,2.07)	1.52(1.04,2.13)	1.60(1.12,2.31)	0.278
LDL, mmol/L (IQR)	1.81(1.37,2.26)	1.95(1.47,2.72)	1.91(1.40,2.68)	<0.001
HDL, mmol/L (IQR)	1.08(0.95,1.24)	1.02(0.88,1.15)	0.97(0.84,1.13)	<0.001
Lp(a), mmol/L (IQR)	13.95(7.50,29.98)	14.60(6.50,32.80)	15.4(7.60,33.20)	0.733
Cr, umol/L (IQR)	68.30(57.88,78.03)	72.40(61.90,83.40)	74.25(63.53,92.25)	<0.001
TyG, (IQR)	8.77(8.46,9.20)	8.89(8.46,9.27)	8.96(8.55,9.44)	0.002
Gensini (IQR)	8.00(4.00,15.12)	34.00(27.00,44.00)	76.00(64.00,96.00)	<0.001

Data are presented as IQR, mean ± SD, or n (%).

Abbreviations: BMI, body mass index; SBP, systolic blood pressure; DBP, diastolic blood pressure; FPG, fasting plasma glucose; TC, total cholesterol; TG, triglycerides; HDL-C, high-density lipoprotein cholesterol; LDL-C, low-density lipoprotein cholesterol; Cr, serum creatinine concentration; TyG, triglyceride-glucose index; Lp(a), lipoprotein(a).

**Fig 1:**
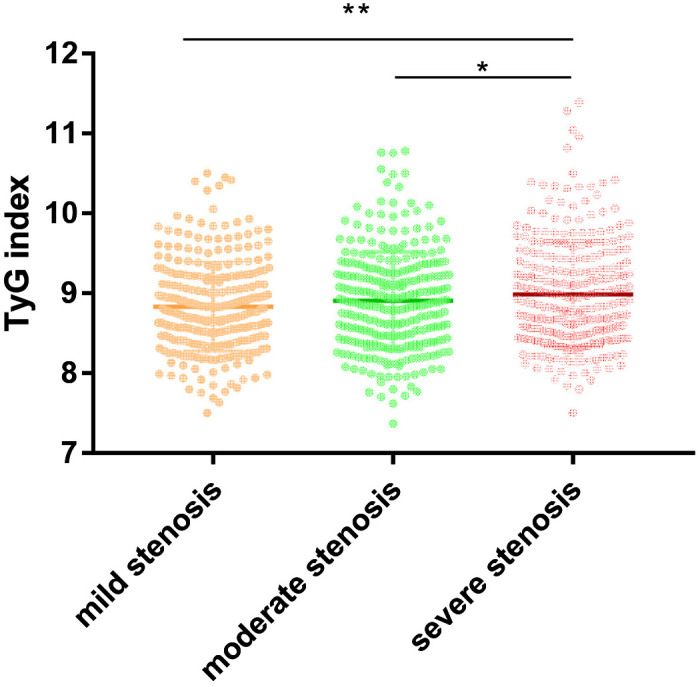
Scatter plots comparing TyG index values among mild, moderate, and severe CAD groups. *P < 0.05, *P < 0.001.

As illustrated in Fig 1, the TyG index differed significantly between the mild and severe stenosis groups (P = 0.0021, **), as well as between the moderate and severe groups (P = 0.0173, *). To further explore the relationship between the TyG index and coronary artery disease (CAD) risk factors, independent associations were examined within subgroups stratified by stenosis severity, as presented in Table 3. In patients with severe stenosis, the TyG index demonstrated significant positive correlations with BMI, Gensini score, TC, TG, and LDL-C. Conversely, HDL-C exhibited a significant negative correlation with the TyG index (r = –0.408, P < 0.001). Notably, the correlation between the TyG index and the Gensini score was stronger in the severe stenosis group than in the moderate group (R = 0.146, P = 0.016).

**Table 3:** Associations between the TyG index and clinical parameters in different stenosis severity groups.

Variable	G1		G2		G3	
	Correiation coefficient(r)	P value	Correiation coefficient(r)	P value	Correiation coefficient(r)	P value
TC,mmol/L	0.352	<0.001	0.298	<0.001	0.286	<0.001
TG,mmol/L	0.900	<0.001	0.907	<0.001	0.875	<0.001
HDL,mmol/L	−0.249	<0.001	−0.305	<0.001	−0.295	<0.001
LDL,mmol/L	0.242	<0.001	0.218	<0.001	0.098	0.106
LPA,mmol/L	−0.132	0.024	−0.034	0.560	−0.159	0.009
FPG,mmol/L	0.447	<0.001	0.539	<0.001	0.550	<0.001
Age,years	−0.076	0.182	−0.263	<0.001	−0.032	0.603
SBP,mmHg	0.031	0.594	0.013	0.820	0.008	0.894
DBP,mmHg	−0.016	0.780	0.160	0.006	0.002	0.980
Cr,mmol/L	0.023	0.692	−0.026	0.660	0.045	0.463
BMI,kg/m²	0.226	<0.001	0.275	<0.001	0.105	0.083
Gensini	−0.002	0.979	0.114	0.049	0.146	0.016

Data are presented as the IQR, mean ±SD or n (%).

Abbreviations: BMI, body mass index; SBP, systolic blood pressure; DBP, diastolic blood pressure; FPG, fasting plasma glucose; TC, total cholesterol; TG, triglycerides; HDL-C, high-density lipoprotein cholesterol; LDL-C, low-density lipoprotein cholesterol; Cr, serum creatinine concentration; TyG, triglyceride-glucose index; Lp(a), lipoprotein(a).

### TyG index and coronary artery lesion severity: Predictive value

Multiple logistic regression analysis (Table 4) was conducted to assess the relationship between the TyG index and the severity of coronary artery stenosis. When treated as a continuous variable, an elevated TyG index was significantly associated with coronary stenosis (odds ratio [OR] = 1.591; 95% confidence interval [CI]: 1.237–1.866). After adjusting for potential confounders—including age, sex, diabetes, and hypertension—the adjusted OR was 1.492 (95% CI: 0.835–2.665).

**Table 4:** Multiple and ordered logistic regression analyses of factors associated with CHD.

	Model1	P	Model2	P	Model3	P
	OR(95%CI)		OR(95%CI)		OR(95%CI)	
TyG	1.519(1.237,1.866)	<0.001	1.414 (1.134,1.762)	0.002	1.492(0.835,2.665)	0.177
Q1	1		1		1	
Q2	0.976(0.689,1.384)	0.893	0.943(0.661,1.344)	0.745	0.932(0.642,1.351)	0.710
Q3	1.147(0.809,1.625)	0.441	1.107(0.766,1.580)	0.574	1.045(0.691,1.581)	0.834
Q4	1.692(1.193,2.401)	0.003	1.481(1.023,2.143)	0.037	1.101(0.605,2.004)	0.753

Model 1: unadjusted; Model 2: adjusted for sex, age, diabetes, and hypertension; Model 3: adjusted for sex, age, diabetes, hypertension, TG, high-density lipoprotein cholesterol (HDL), low-density lipoprotein cholesterol (LDL), serum creatinine, and blood glucose levels. CI denotes confidence interval; OR denotes odds ratio; TG, triglycerides; HDL, high-density lipoprotein cholesterol; LDL, low-density lipoprotein cholesterol.

**Fig 2:**
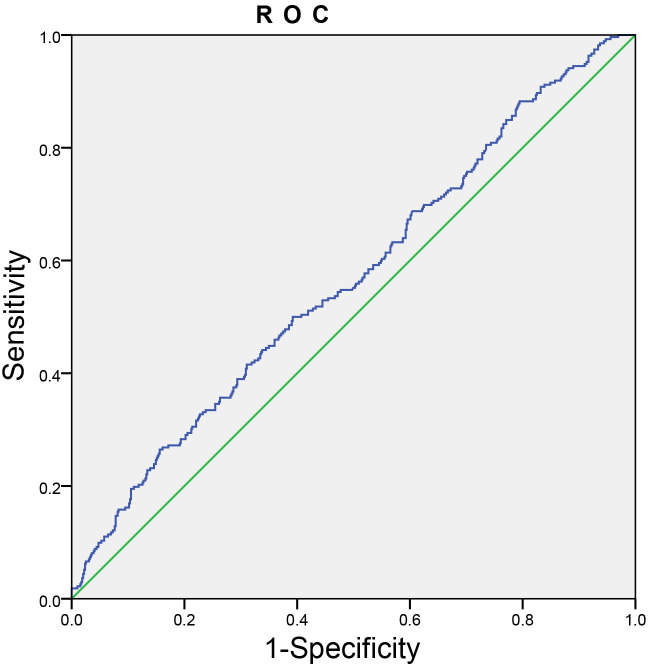
ROC curve of the TyG index in the detection of coronary artery disease.

We subsequently investigated the association between the severity of coronary artery lesions and the TyG index. After adjusting for covariates including age, sex, diabetes, and hypertension, individuals in the second, third, and fourth quartiles of the TyG index demonstrated a higher incidence of severe coronary stenosis compared to the reference group (OR = 0.932; 95% CI: 0.642–1.351; OR = 1.045; 95% CI: 0.691–1.581; OR = 1.101; 95% CI: 0.605–2.004). Fig 2 presents the ROC curve for the TyG index, which may serve as a potential predictive marker for identifying patients with significant coronary stenosis. The area under the ROC curve (AUC) was 0.567 (95% CI: 0.525–0.608; p = 0.002), with corresponding sensitivity and specificity values of 26.5% and 84.4%, respectively.

## Discussion

This study aimed to identify predictive markers for early assessment of CHD risk through a retrospective analysis of 861 patients who underwent coronary angiography, with a specific focus on evaluating the TyG index as a predictor of severe vascular stenosis in individuals with CHD. Our findings revealed that patients with moderate to severe CHD exhibited significantly higher TyG index levels compared to those with mild CHD. Elevated TyG index values were positively associated with increased severity of coronary artery stenosis. ROC curve analysis—including evaluation of the area under the curve, sensitivity, and specificity—indicated that the TyG index may serve as a diagnostic indicator for the severity of coronary artery stenosis and has potential utility as a predictive biomarker.

Previous studies have reported a potential linear relationship between the TyG index and the incidence of cardiovascular disease, suggesting that elevated TyG levels may be associated with an increased risk of cardiovascular events [23]. Other investigations have emphasized the importance of early intervention by monitoring TyG levels and long-term lifestyle behaviors to reduce cardiovascular risk [24]. Generally, a positive correlation is observed between age and the TyG index, as advancing age is often accompanied by increased insulin resistance, leading to elevated triglyceride and fasting glucose levels. However, our study identified an inverse relationship between age and the TyG index. This divergence may be attributed to lifestyle interventions such as exercise and dietary modifications, which improve insulin sensitivity and may reduce the TyG index, thereby concealing the age-related risk.

Several studies have established hyperlipidemia as a key risk factor in the pathogenesis of cardiovascular disease [8,25–28]. In this context, our study investigated the relationship between the TyG index and traditional cardiovascular risk factors. The TyG index showed a positive correlation with TC and TG, while a negative correlation was observed with high-density lipoprotein cholesterol (HDL). These results are consistent with previous studies, which demonstrated that CHD patients with elevated TyG index values typically exhibit increased total plasma cholesterol and reduced HDL-C levels [29,30]. Furthermore, many patients with CHD undergo standardized pharmacological treatment to manage lipid and glucose levels, which may lead to reduced TyG values and explain deviations from previously reported trends. Our investigation underscores the significance of the TyG index as a biomarker for cardiovascular health and its potential role in assessing the severity of coronary artery stenosis.

Additionally, we explored the predictive performance of the TyG index across different quartile classifications. Multivariate regression analysis revealed that higher TyG index levels were significantly associated with increased coronary artery stenosis severity. This relationship remained robust prior to adjusting for confounders. However, after controlling for serum lipid levels (TG, HDL, LDL), the association between the TyG index and stenosis severity was attenuated. This finding highlights the critical role of lipid metabolism in coronary artery calcification and underscores the necessity of accounting for lipid parameters in predictive models evaluating the TyG index.

Previous research has reported that individuals in the upper quartiles (Q3 or Q4) of TyG levels are at increased risk of cardiovascular disease [31,32]. Our results confirmed a similar association between the fourth quartile of TyG and the severity of coronary artery stenosis. However, after adjusting for serum lipid levels, this association diminished, suggesting that lipid parameters are important confounders influencing the observed relationship. Notably, the diagnostic significance of the TyG index appeared to be more pronounced in patients with moderate coronary stenosis. The correlation between the TyG index and LDL was also more prominent in patients with mild to moderate stenosis and was not statistically significant in those with severe stenosis.

It is important to acknowledge that information regarding statin use was unavailable for a considerable number of participants and, therefore, was not included in the multivariate regression analysis. This omission may introduce bias regarding the impact of lipid-lowering therapy on coronary artery stenosis severity. Furthermore, the lack of follow-up data limits our ability to assess long-term clinical outcomes. Nevertheless, previous literature suggests that the TyG index has prognostic value for predicting cardiovascular events and outcomes [33,34]. Future research will aim to incorporate longitudinal follow-up data to further validate the predictive utility of the TyG index.

Coronary angiography is an invasive procedure employed to assess the extent of coronary atherosclerosis by measuring various blood parameters indicative of CHD severity. Consequently, identifying novel blood-based biomarkers that fulfill clinical diagnostic needs and offer reliable, less invasive alternatives is of paramount importance. In this context, we conducted a comprehensive evaluation of CHD progression by examining parameters associated with glucose and lipid metabolism. Blood testing provides a non-invasive method to predict CHD development and may facilitate more appropriate application of clinical interventions.

Correlation analysis suggests that the TyG index is associated with several established risk factors for CHD. One study reported a significant correlation between BMI, TC, HDL-C, LDL-C, and the Gensini score (r > 0.2, p < 0.05) [35]. Another investigation indicated that the TyG index positively correlates with FBG, TC, TG, and LDL-C, while showing a negative correlation with estimated glomerular filtration rate (eGFR), left ventricular ejection fraction (LVEF), and HDL-C. However, no significant associations were identified between the TyG index and age, BMI, SBP, DBP, uric acid (UA), or the Gensini score [36].

The TyG index is widely recognized as a useful biomarker for assessing metabolic risk and plays a valuable role in the early identification of coronary heart disease. The Gensini score, by contrast, is considered the gold standard for quantifying the severity of coronary artery lesions post-diagnosis. The integration of these two indicators enables a more comprehensive evaluation of CHD by concurrently addressing metabolic dysfunction and vascular pathology. This synergistic approach not only enhances diagnostic accuracy but also contributes meaningfully to the refinement of research methodologies in cardiovascular medicine.

Several limitations of this study should be acknowledged. First, the study focused exclusively on CHD severity, as all participants were diagnosed with CHD, and no control group was included for comparative analysis. Second, potential confounding variables such as dietary habits and medication use were not incorporated into the analysis. Third, the cross-sectional design precludes the ability to infer causality between TyG levels and the risk of coronary artery calcification; future longitudinal studies are needed to establish this relationship. Fourth, the study focused solely on the TyG index without comparing it to related composite metrics such as TyG/HDL-C, TyG-BMI, TyG-WC, or TyG-WHtR. Future research should explore the associations between these indices and various cardiovascular diseases. Lastly, the study population was derived from a single geographic region with a relatively limited sample size. Hence, validation through multicenter and multiregional studies involving larger populations is necessary. Additionally, only initial laboratory test results obtained upon admission were analyzed in this study, which may introduce selection bias that warrants further investigation.

## Conclusion

In conclusion, our findings indicate that the TyG index is a valuable biomarker for assessing the severity of CHD in clinical settings. It may serves as an independent prognostic indicator of disease severity, however, this association requires validation in prospective longitudinal studies. A significant association was observed between the TyG index and the extent of coronary artery stenosis. Owing to its practicality and cost-effectiveness, the TyG index holds considerable promise for implementation in primary healthcare settings across China, thereby supporting effective risk stratification and targeted intervention strategies.

## Supporting information

S1 Table The Gensini scoring system utilized in this study. (DOCX)

S2 Table The original data are provided and available in the supplementary file, S2 Table. (XLSX)
