# Supplementary material for: Triglyceride-glucose index is associated with the severity of coronary artery disease
Source: PLoS One. 2026 Aug 17;21(8):e0356426. doi: 10.1371/journal.pone.0356426 (PMC13480658; doi:10.1371/journal.pone.0356426)
Supplement: Supplementary file 1 [file pone.0356426.s001.docx]

TableS1

| Stenosis | score | Lesion | score |
| --- | --- | --- | --- |
| 1%-25% | 1 | LM | 5 |
| 26%-50% | 26 | Proximal LAD or LCX | 2.5 |
| 51%-75% | 4 | Middle LAD | 1.5 |
| 76%-90% | 8 | Distal LAD | 1.0 |
| 91%-99% | 16 | Middle or distal LCX | 1.0 |
| 100% | 32 | RCA | 1.0 |
|  |  | D1 | 1.0 |
|  |  | OM1 | 1.0 |
|  |  | Small branch | 0.5 |

LM, left main; LAD, left interior descend; LCX, left circumflex; RCA, right coronary artery; D1, First diagonal branch; OM1, First cuspal lobe. The Gensini score for each affected segment is equal to the stenosis score multiplied by the lesion factor.
